# The complex evolutionary history of big-eared horseshoe bats (*Rhinolophus macrotis* complex): insights from genetic, morphological and acoustic data

**DOI:** 10.1038/srep35417

**Published:** 2016-10-17

**Authors:** Keping Sun, Rebecca T. Kimball, Tong Liu, Xuewen Wei, Longru Jin, Tinglei Jiang, Aiqing Lin, Jiang Feng

**Affiliations:** 1 Jilin Provincial Key Laboratory of Animal Resource Conservation and Utilization, Northeast Normal University, Changchun, China; 2 Department of Biology, University of Florida, Gainesville, Florida, United States of America

## Abstract

Palaeoclimatic oscillations and different landscapes frequently result in complex population-level structure or the evolution of cryptic species. Elucidating the potential mechanisms is vital to understanding speciation events. However, such complex evolutionary patterns have rarely been reported in bats. In China, the *Rhinolophus macrotis* complex contains a large form and a small form, suggesting the existence of a cryptic bat species. Our field surveys found these two sibling species have a continuous and widespread distribution with partial sympatry. However, their evolutionary history has received little attention. Here, we used extensive sampling, morphological and acoustic data, as well as different genetic markers to investigate their evolutionary history. Genetic analyses revealed discordance between the mitochondrial and nuclear data. Mitochondrial data identified three reciprocally monophyletic lineages: one representing all small forms from Southwest China, and the other two containing all large forms from Central and Southeast China, respectively. The large form showed paraphyly with respect to the small form. However, clustering analyses of microsatellite and Chd1 gene sequences support two divergent clusters separating the large form and the small form. Moreover, morphological and acoustic analyses were consistent with nuclear data. This unusual pattern in the *R. macrotis* complex might be accounted for by palaeoclimatic oscillations, shared ancestral polymorphism and/or interspecific hybridization.

Species are not only the basic taxonomic unit, but also one of the most important units of interaction between ecology and evolution. Species delimitations and their distributions have traditionally been through morphological traits^1^. However, not all evolutionary changes show plainly as observable phenotypic traits^2^. Complex geographic landscapes, palaeoclimatic oscillations, as well as biological and ecological traits of organisms often lead to complex population structure and speciation processes (e.g. refs 3, 4 and 5). Unravelling those evolutionary mechanisms and processes becomes increasingly important because it is crucial to understanding speciation, from the earliest stages of incipient speciation through complete reproductive isolation (e.g. refs 6 and 7).

In some taxa with complex evolutionary histories, unexpectedly widespread species-level paraphyly and polyphyly in mitochondrial (*mt*) DNA analyses have been reported^6^,^7^,^8^,^9^, suggesting conflicting geographic patterns between mtDNA and nuclear (nc) DNA or phenotype. Except for cases of imperfect taxonomy^8^, these prevalent instances of paraphyly uncovered by mtDNA in animals are primarily attributed to incomplete lineage sorting and/or introgressive hybridization^8^,^9^,^10^. Incomplete lineage sorting produces a failure of mtDNA haplotype fixation from ancestral polymorphisms when speciation events are short and ancestral effective population sizes are large. Alternatively, hybridization can lead to mitochondrial capture, such that the mitochondrial and nuclear genomes yield different histories. Natural hybridization among closely related animal species has been increasingly uncovered in recent years using genotypic data^11^, making this an important alternative hypothesis to consider.

Bats represent over 20% of mammal species^12^. However, documented cases of complex evolutionary histories involving paraphyly in this group are very rare^13^,^14^,^15^,^16^,^17^. Complex evolutionary histories are relatively common in birds (e.g. refs 8 and 10), and since bats have similar vagility to birds, the limited number of observations in bats may be due to limited studies. *Rhinolophus* is one of the most diverse and complex bat genera. We recently explored the taxonomy of big-eared horseshoe bats (*Rhinolophus macrotis*) based on sampling in one province. Since the large and small form are sympatric, and have significant differences in both morphology (size) and echolocation frequencies (~16 kHz) as well as a relatively high mitochondrial cytochrome *b* (Cyt*b*) divergence (3.16–3.25%), this is a species complex with cryptic species^18^. This species complex is widely distributed in Southeast Asia, and their morphology and echolocation frequency are extensively variable^19^. However, based on our recent field surveys in China, we observed the species complex had a continuously and widespread distribution throughout Central and South China, and they frequently roost in colonies including both small and large forms (Fig. 1), which suggests that complex evolutionary processes might be occurring in the *R. macrotis* complex.

In this study, in order to examine their evolutionary history and speciation, we used ten unlinked genetic markers, including mtDNA (Cyt*b* and control region) and ncDNA (eight microsatellite loci and Chd1 gene) to reconstruct the phylogeny of the big-eared horseshoe bat species complex from China. We combined this with morphological data (i.e. external measurements) and acoustic data (i.e. echolocation frequency). Our study aimed to (i) determine genetic lineages in this species complex; (ii) determine whether the mtDNA variation is congruent or conflicts with the nuclear and phenotypic data (i.e. morphological and acoustic measurements); (iii) if conflicting, evaluate potential mechanisms leading to patterns within this species complex; (iv) and, evaluate the effect of palaeoclimatic changes on this species complex.

## Results

### Sequence characteristics

For 79 individuals in the *R. macrotis* complex sampled from 14 localities across the entire Chinese range of the species, we found much variation in Cyt*b* (1,140 bp) and control region (464 bp). A total of 29 haplotypes of Cyt*b* were defined based on 103 polymorphic sites (71 of which were parsimony informative). No insertions or deletions were found. Fifty haplotypes of the control region were defined based on 115 polymorphic sites (90 of which were parsimony informative). The alignment of the combined Cyt*b* and control region (1,604 bp) resulted in 53 haplotypes. For the autosomal Chd1 gene (742 bp), a total of 20 haplotypes were defined from 71 individuals based on 24 polymorphic sites (6 of which were parsimony informative).

### Mitochondrial trees and genetic divergence

**Figure 1:**
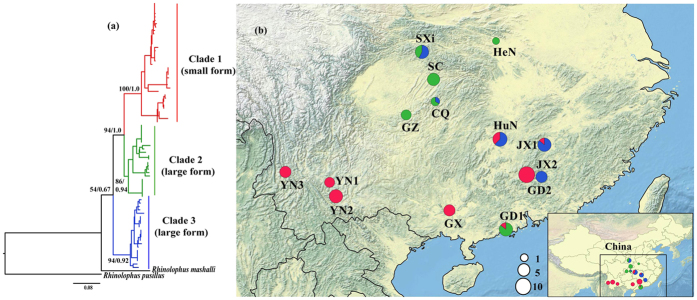
Mitochondrial gene tree and sample sites in this study. (**a**) Phylogenetic tree based on the analysis of the combined mitochondrial Cyt*b* and control region. Numbers above the branches are bootstrap support from in Maximum likelihood followed by posterior probabilities from Bayesian analyses. (**b**) Sampling sites for the *mt* lineages included in the study. Circle sizes are proportional to the number of individuals captured. Abbreviations of samples populations are indicated in Table S1. The map was made with QGIS 2.8 (http://www.qgis.org) and Natural Earth public domain map data (http://www.naturalearthdata.com/about/terms-of-use/), and modified in Illustrator.

Maximum likelihood and Bayesian phylogenetic reconstruction based on the combined mtDNA data (Fig. 1a) and on the separate mtDNA genes (Figs S1 and S2, Supporting Information) resulted in similar tree topologies in which three well-supported clades were recovered. Clade 1 contained all small forms of *R. macrotis* from Southwest China, including those small forms identified previously^18^. Clades 2 and 3 contained all large forms of *R. macrotis* from Central and Southeast China, respectively. The large forms identified in Sun *et al.*^18^ were all contained in Clade 3. No haplotype was shared among the three clades.

The ranges of the three clades were partially sympatric (Fig. 1b). Both SXi and CQ populations contained individuals from Clades 2 and 3 (all large forms), both the HuN and JX1 populations contained individuals from Clades 1 and 3, and the GD1 population contained individuals from Clades 1 and 2.

In general, intraspecific genetic divergence levels among bats are typically less than 2% within Cyt*b*^20^. Thus we used Cyt*b* to calculate the intra-lineage and inter-lineage genetic differences. In this species complex, the maximum level of uncorrected intra-clade divergence was ≤2% (Table 1). Among the three clades, the minimum uncorrected divergence was over 2% (Table 1), though never exceeded 4%.

Since the clades were distinct genetic pools, population genetics analyses were performed within each lineage. Genetic diversity was highest in Clade 1, followed by Clades 3 and 2 for both the Cyt*b* and control region (Table 2).

### Demographic inference

We estimated divergence times among clades using Cyt*b* (Table 3). The inferred TMRCA for all *R. macrotis* complex was 1.51 Ma (Million years ago), with similar divergence times *mt* Clades 1 and 2 (0.64 and 0.66 Ma, respectively). In contrast, *mt* Clade 3 originated much later. Tajima’s D values and Fu’s Fs tests indicated that none of the three *mt* lineages deviated significantly from neutrality (Table 4). Unlike in *mt* Clades 2 and 3, analysis of *mt* Clade 1 failed to reject the model of population expansion (Table 4), with an estimated timing of expansion occurring 78.8 Ka (Thousand years ago) (95% CI: 38.1–96.1 Ka).

### Nuclear genetic analyses

Based on microsatellites and the Chd1 gene, the large form populations generally showed higher genetic diversity than the small form (Table 3, Table S2, Supporting information). All eight microsatellite loci were polymorphic within each form, and only 3.4% of 120 population-locus combinations significantly deviated from HWE. No sign of linkage disequilibrium was detected. Micro-checker identified a potential of 3.8% null alleles in population-locus tests. However, null alleles and deviations from HWE were not associated more frequently with any particular locus. Therefore we retained all loci for subsequent analyses.

**Figure 2:**
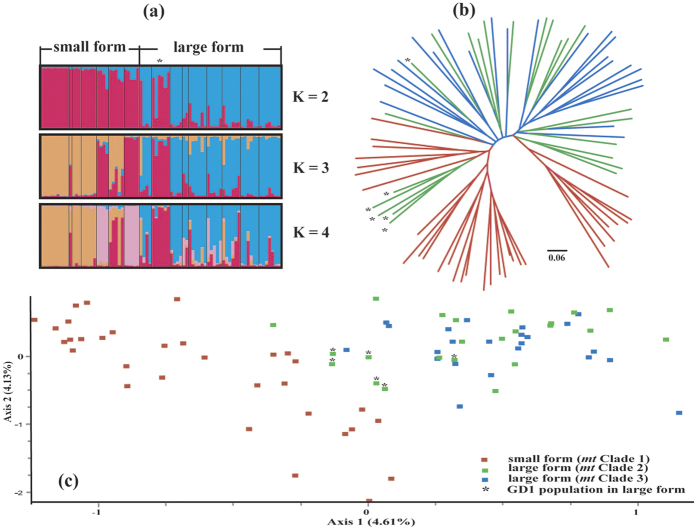
Individual clustering based on the analysis of microsatellite data. (**a**) Bayesian cluster analyses with Structure (*K* = 2, 3, 4) of all samples based on eight microsatellites. Each vertical bar represents one individual and its probability of being assigned to a cluster. (**b**) Unrooted neighbor-joining tree reconstructed from Nei *et al.*^63^ D_A_ distances based on microsatellite genotypes of all individuals. Colours indicate the membership of each *mt* lineage. (**c**) Factorial corresponding analysis based on the microsatellite data. All the asterisks represent the individuals from GD1 population in the large form.

Using the Evanno criterion with structure output suggested that *K* = 2 was most likely, with the large form and small form largely identified as distinct clusters. The exception was one large form population (GD1; Fig. 2a) in which only one individual was correctly identified as the large form, while the other five were assigned to the small form. For *K* = 3 or 4, no new clusters were detected in the large form, but some cryptic genetic structure was found in the small form. Similar results were obtained from the NJ tree, which demonstrated that the small forms made two clades, rather than a single clade (Fig. 2b), while the large form individuals from *mt* Clade 2 and *mt* Clade 3 did not form independent monophyletic groups and were instead scattered throughout the tree. In the FCA (Fig. 2c), all small forms were clearly identified as a separate genetic group, while large forms from *mt* Clades 2 and 3 were intermixed. Moreover, the specimens from GD1 population of large form were located between the small and large forms in this analysis. Results from GENELAND (Fig. S3, Supporting Information), which can detect population structure in relation to geographic and genetic information, also supported the existence of two main genetic clusters associated with the small form and the large form, except for GD1 and HuN populations, where the two forms are sympatric. In three independent runs, GD1 and HuN populations were inferred to cluster with the small form and the large form (Fig. S3a,b), respectively, while the opposite result occurred in the other two runs (Fig. S3c,d).

**Figure 3:**
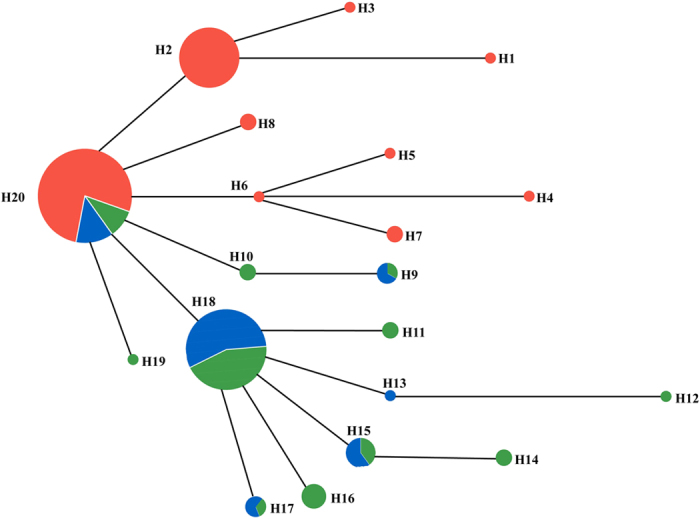
Haplotype network of *Rhinolophus macrotis* species complex samples based on nuclear Chd1 gene sequences. Colours indicate the membership of each *mt* lineage, including Clade 1 (red), Clade 2 (green) and Clade 3 (blue).

Consistent with the microsatellite results, the large form individuals from *mt* Clade 2 and *mt* Clade 3 did not form independent Chd1 haplogroups and instead shared five haplotypes (Fig. 3). However, the large form and the small form had their own distinct haplotypes with one exception (Fig. 3).

**Figure 4:**
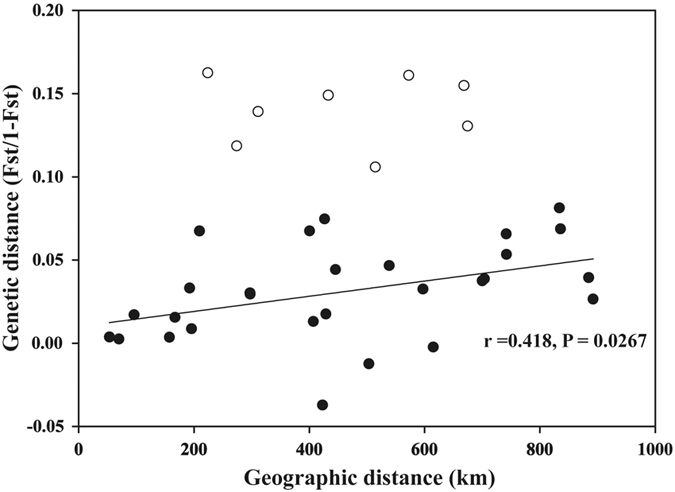
Regression of genetic distance based on microsatellite data versus geographic distances for pairwise population comparisons in large forms including *mt* Clades 2 and 3. White circles represent the distances between GD1 population in large forms and other populations.

We focused on the geographic pattern of nuclear genetic variation within *mt* Clades 2 and 3, as the above analyses failed to detect their genetic differentiation in individual clustering (Figs 2 and 3). No significant IBD was found within the large forms (*P* > 0.05). However, when excluding the GD1 population, the test of IBD suggested that the two lineages had not evolved separately but were connected because smooth clinal variation rather than a geographic break was observed in the nuclear genetic variation (Fig. 4).

### Phenotypic differences

**Figure 5:**
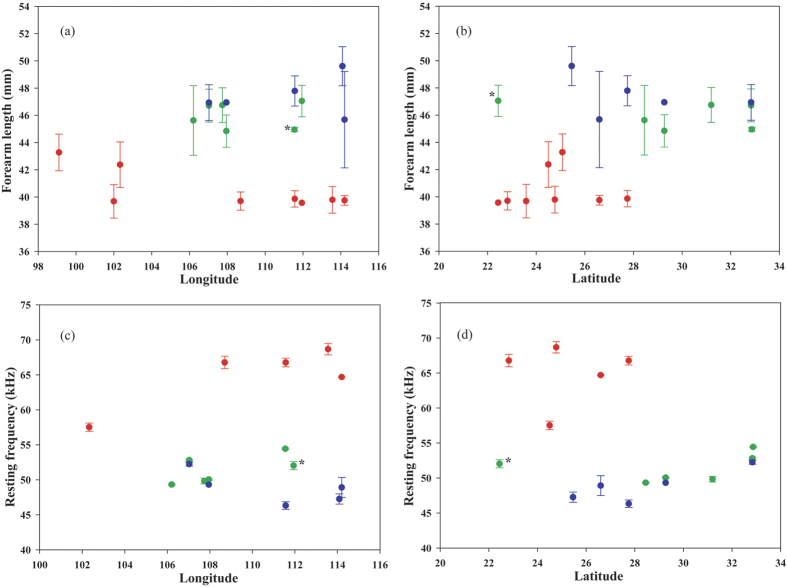
The variation of forearm length and resting frequency in echolocation calls among the large form and the small form of *R. macrotis* species complex. Circles represent the mean values. Colours indicate the membership of each *mt* lineage, including Clade 1 (red), Clade 2 (green) and Clade 3 (blue). The asterisks represent the individuals from GD1 population in large forms.

Phenotypic analyses distinguished the large and small forms using either forearm length or resting frequency of echolocation calls (Fig. 5), including in sympatric populations.

Within the large form, morphological differentiation was not significant between *mt* Clade 2 and *mt* Clade 3. Forearm length was significantly explained by site, but not by sex and *mt* lineage, or by interactions among variables (Table 5). Resting frequency was significantly explained by sex and *mt* lineage, as well as by site. No significant interaction was found between effects (Table 5).

We focused on the geographic pattern of acoustic variation within *mt* Clade 2/Clade 3 of the large form as we found no significant morphological differences between these two clades. We detected positive significant associations between resting frequency and both longitude (r = 0.39, *P* = 0.0011) and latitude (r = 0.284, *P* = 0.0199). When excluding GD1, clinal variation was much more significant (longitude: r = 0.48, *P* < 0.0001; RF vs latitude: r = 0.74, *P* < 0.0001).

## Discussion

This study highlights the complex evolutionary history of big-eared horseshoe bats in China, and adds to the limited literature documenting complex evolutionary histories in bats. We document here the incongruence between *mt* and nuclear genetic structures. Three *mt* lineages were identified: Clade 1 (small form) and Clade 2 (large form), which are closely related and separated by short genetic distances, and the more distantly related Clade 3 (large form). In contrast, only two genetic entities were identified on the basis of nuclear data: small form (*mt* Clade 1) and large form (combined *mt* Clades 2 and 3). The morphological and acoustic data were consistent with the nuclear genetic data, as they also did not separate the *mt* Clade 2 and *mt* Clade 3 lineages. These results suggest the occurrence of complex processes during the evolution of the big-eared horseshoe bats in the study area, such as palaeoclimatic changes, allopatric speciation, incomplete lineage sorting and/or genetic introgression.

### The effect of palaeoclimatic changes and allopatric speciation of the small form

Pleistocene climatic fluctuations and accompanying ecological changes in China have presented severe challenges to the survival of bat species, greatly affecting their population differentiation and contemporary distribution^3^,^21^,^22^. In this study, the TMRCA of the entire *R. macrotis* complex populations dated to 1.51 Ma. During this period, China was experiencing glacial-interglacial cycles, including the Poyang glacial stage (1.8 Ma) and the Dagu glacial stage (1.1 Ma)^23^. Climatic changes and temperature decline may have forced the big-eared horseshoe bats to diverge from other bat species and evolve separately.

The TMRCAs of the combined *mt* Clade 1 (small form) and *mt* Clade 2 (large form) dated to 1.16 Ma, suggesting that the two *mt* lineages differentiated from each other between 1.16–0.64 Ma. This time was consistent with two major cold glacial stages, Xixiabangma glaciations (1.17–0.8 Ma)^24^ and the Naynayxungla glaciations (0.71–0.59 Ma)^25^. The former stage is one of the coldest recent climatic periods based on a high mass accumulation rate for Chinese loess^26^. At that time, the populations might have become isolated into different refugia and occupied different habitats and climate zones either due to, or eventually leading to, local ecological adaptation, and two distinct lineages distributed in Central and Southwest China.

Despite this recent divergence, there is strong evidence to suggest that the small form is genetically isolated from the large form (*mt* Clades 2 and 3). The small forms clearly formed a distinct genetic pool on the basis of nuclear genetic analyses (Figs 2 and 3). Moreover, both the morphological and acoustic data showed an obvious differentiation between small and large forms, even for sympatric populations (Fig. 5), consistent with those found in Sun *et al.*^18^. Given their current geographic distribution (Fig. 1), it seems possible that small forms diverged from the widely distributed large forms during glaciations, which would represent a case of allopatric or peripatric speciation. However, for peripatric speciation, an expectation might be that estimates of the genetic diversities of the small form would be lower than in the large forms due to a founder effect. Since all estimates of the small form (haplotype and nucleotide diversities for mtDNA, allelic richness for microsatellites, and genetic diversity for the Chd1) were similar between the small and large forms (Table 2), allopatric speciation is more likely.

### Deep mitochondrial divergence in the large form

The mitochondrial genetic analyses indicated that large forms diverged into two distinct lineages, Clade 2 and Clade 3 with a level of divergence similar to that between the small and large forms. Additionally, these two *mt* clades show distinct geographic distribution, with Clade 2 primarily occurring Central China, whereas Clade 3 is primarily in Southeast China (Fig. 1), although there is sympatry in two localities (CQ and SXi). These results suggest a cryptic mitochondrial lineage in the large form in China.

However, it was not possible to differentiate between the specimens of the two *mt* lineages on the basis of nuclear microsatellite and Chd1 data, morphological measurements or acoustic signals. When considering sites, we detected variation in forearm length and call resting frequency, though there was no significant relationship for *mt* lineage. Additionally, in this study, we found no distinction in call frequency in the sympatric populations, CQ and SXi. For instance, the mean values of resting frequency were 50.05 kHz in *mt* Clade 2 and 49.32 kHz in *mt* Clade 3 within CQ, and 52.82 kHz in *mt* Clade 2 and 52.26 kHz in *mt* Clade 3 within SXi (Table S3, Supporting information). This phenomenon is also found in *Pipistrellus kuhlii*^27^, which has two divergent mitochondrial lineages, but similar nuclear genotypes in Western Europe.

We did detect a clinal central-southeast variation in call frequency (with frequency gradually decreasing with longitude, but increasing with latitude). The observed pattern of geographic variation could be due, at least in part, to cultural drift. In general, cultural transmission and copying errors are major drivers of stochastic divergence in learned vocal signals^28^,^29^, including in some bat species, i.e. *Rhinolophus ferrumequinum*^30^, *Hipposideros armiger*^31^, *Rhinolophus pumilus*^32^. However, environmental factors in call clinal variation could not be ruled out. The smooth cline rather than a geographic break in acoustic variation suggests the occurrence of regular gene flow between the two *mt* lineages in large forms.

### Large form mitochondrial gene-tree paraphyly relative to small form

An increasing number of studies have reported mitochondrial species-level paraphyly (e.g. refs 6, 7 and 8). In our study, phylogenetic analyses revealed that *mt* Clade 1 (small forms) was nested within the *mt* Clades 2 + 3 group (large forms), rendering large forms paraphyletic with respect to small forms. Two evolutionary processes may account for this mitochondrial paraphyly: (i) incomplete lineage sorting and the persistence of highly divergent *mt* lineages, and (ii) mitochondrial introgression.

The persistence of highly divergent *mt* lineages within a species may reflect maintenance of two lineages in a panmictic population with a large effective population size^33^. Since genetic drift is weak in very large populations, this leads to increasing coalescence times and the probability of observing the persistence of highly divergent lineages. Moreover, the greater spatial homogenization of the nuclear genome relative to the *mt* genome (such as we observed) can also occur after two *mt* lineages come into contact. Since most bat species have female philopatry and male-biased dispersal^34^, highly divergent *mt* lineages may spread more slowly than the nuclear genome, as occurs in the big brown bat (*Eptesicus fuscus*)^35^. We determined that the current clinal north-south distribution of *mt* Clades 2 and 3 was likely a consequence of isolation in different Pleistocene refugia, followed by range expansion and the establishment of a secondary contact zone. Thus, retention of unique *mt* lineages that arose in isolation, followed by homogenization of the nuclear genome, is possible in the *R. macrotis* complex.

Alternatively, high mtDNA divergence could also be caused by hybridization with a closely related species, leading to introgression of mtDNA^9^,^36^. If this is the case, then we have presumably sampled the nuclear genome of a single species, together with its *mt* genome plus an introgressed *mt* genome. This scenario implies that another unsampled species with a different nuclear genome and one of the *mt* lineages identified in our data set exists elsewhere in China. One of individual from JX1 population in *mt* Clade 3 had 0.79–1.76% mitochondrial Cyt*b* divergence with an unidentified *Rhinolophus* species, *Rhinolophus sp1.*^37^, suggesting that introgression of the *mt* genome may have occurred. We reconstructed trees using all of our data with the published Cyt*b* sequence of *R. sp1.* (Fig. S4, Supporting Information), which showed that *R. sp1.* clustered within *mt* Clade 3. This result suggested that *mt* Clade 3 may have an introgressed *mt* genome from *R. sp1.* Certainly, sampling efforts should focus on population JX1 to sample more *R. sp1.* in the future. Alternatively, the ‘unsampled species’ may have gone extinct after ancient hybridization events. If so, its *mt* lineage now only survives in its new host, and a process of competitive replacement of mtDNA has occurred, with no nuclear introgression or morphological changes, such as has been suggested in some other animals, i.e., hare^38^ and lizard^39^.

### Secondary contact between small form and large form

In our previous study^18^, the large and small forms were identified from sympatric large-eared horseshoe bats in China, suggesting the existence of a cryptic species. In this study, three sympatric populations (GD1, HuN and JX1) were detected between small and large forms (Fig. 1), suggesting secondary contact after putative speciation.

Based on our analyses of population demographic history, the small form might have undergone post-glacial range expansion after the Baiyu (the Last) Glaciation^24^, following secondary contact with the large forms. In the mitochondrial trees, all individuals from the three sympatric areas diverged recently (see the top of *mt* Clade 1; Figs S1 and S2). Moreover, the number of small forms is less than large forms in three sympatric areas (Fig. 1), suggesting the small form may be expanding into areas occupied by the large form.

For the HuN and JX1 populations, the individuals were assigned to the correct forms based on microsatellite NJ tree, FCA and Bayesian clustering, suggesting a recent secondary contact between two forms or the maintenance of reproductive isolating barriers in two populations. However, for the GD1 population, only one individual was correctly assigned to the large form group, but five individuals were genetically assigned to the small form group (Fig. 2), though their morphological and acoustic data were obviously different from those of the small form (Fig. 5). Furthermore, the different GENELAND assignments for the GD1 and HuN populations during five independent runs (Fig. S3, Supporting Information) emphasizes the complexity of these two sympatric areas. Such discordance between ncDNA and phenotypic patterns in GD1 might suggest hybridization followed by backcrossing to the small form at this site, such that the number of microsatellite loci we sampled was insufficient to separate large and small forms^40^. If hybridization between the small and large forms has occurred, it is expected that hybrids may have intermediate phenotypes, at least for several generations. In our study, the acoustic data of GD1 may support this hypothesis. When excluding GD1, call frequency was more significantly correlated with longitude in large forms (Fig. 5), suggesting that the call frequency of GD1 obviously deviated from the clinal variation of other large forms (Fig. 5d). Additionally, when excluding GD1, the microsatellite genetic distance is significantly related with geographic distance (Fig. 4), suggesting different genetics between GD1 and other large forms.

## Conclusion

In summary, the evolutionary history of the *R. macrotis* complex appears to be complex. This complexity has led to strong incongruence between *mt* genetic structure, and that from nuclear loci, morphology and acoustic patterns in this species complex. The divergence between the small and large form likely occurred in allopatry due to isolation of populations during Pleistocene glaciation events, and current areas of sympatry appear to be due to more recent secondary contact (likely driven by range expansion of the small form). Current hybridization may be occurring in one sympatric population, though there appears to be reproductive isolation between the small and large forms in other sympatric populations. The occurrence of two highly divergent *mt* lineages in large forms conflicts with the nuclear plus phenotypic data (i.e. morphological and acoustic measurements), which might be due to the persistence of highly divergent *mt* lineages, geographically structured during Pleistocene glaciations, and/or genetic introgression from a species yet to be identified. While such complex patterns have been observed frequently in birds, they have rarely been reported in bats, which are also quite vagile. Our observations add to several other studies on bats, and suggest that the limited studies in bats (relative to birds) may be due to the paucity of studies on bats rather than a fundamental difference in their evolutionary histories. Overall, our results suggest that additional studies in bats may help highlight other cases of complex evolutionary histories.

## Materials and Methods

### Taxa

In China, 79 individuals of big-eared horseshoe bat complex were sampled during 2006–2012 from 14 localities (Fig. 1 and Table S1, Supporting Information), including the 12 specimens of the 7 small form and 5 large form bats published previously^18^. All bats were identified following Csorba *et al.*^19^.

Sampling sizes were different for morphological characters, echolocation calls, mtDNA, microsatellite DNA and the Chd1 gene. For morphological characters (forearm length), 133 independent samples were measured. For echolocation calls, 109 independent samples were recorded. From those individuals with morphological and echolocation data, 79 samples were sequenced for mtDNA, 78 and 71 were examined using microsatellite DNA and Chd1 analyses, respectively. For each sampling locality, we determined the latitude and longitude using GPS (eTrex Vista) and then calculated the geographic distance matrixes among localities.

### Genetics

#### DNA extraction and sequencing

We used a UNIQ-10 column animal genomic DNA isolation kit (Sangon, China) to extract total genomic DNA from collected bat wing membranes which had been preserved in 99% ethanol.

We sequenced two mtDNA fragments: the complete Cyt*b* gene (1,140 bp) and 464 bp of the control region. The complete Cyt*b* fragment for 67 individuals was amplified using primers L14724 and H15915^41^,^42^, which were combined with 12 sequences reported by Sun *et al.*^18^ to obtain the final data set (Table S1, Supporting Information). PCR and sequencing protocols were the same as those described in Sun *et al.*^18^. The fragment of mitochondrial control region for all 79 individuals was amplified using primers P and E, and the PCR methods described in Wilkinson and Chapman^43^. Amplified products were purified and sequenced by Shanghai Sangon Biotechnology Co., Ltd. All Cyt*b* and control region haplotypes of *R. macrotis* complex were deposited in GenBank with accession numbers KX261888-KX261916 and KX261917- KX261966, respectively. All sequences were aligned using GENEIOUS PRO (v. 5.6.6)^44^ and revised manually.

Eight microsatellite loci, Reffer 15, 22, 24, 27, H3, PD3, A4 and PH69A, were amplified using fluorescently labeled primers for 78 individuals. Primer sequences and PCR conditions followed those of Rossiter *et al.*^45^ and Hua *et al.*^46^. Amplified PCR products were analyzed using the ABI 3730 automated DNA sequencer. The resulting sequences were analyzed using GeneMapper ID 3.2 (Applied Biosystems). All loci were screened for null alleles and large allele dropouts using Micro-Checker v2.2.3^47^. Tests for deviation from Hardy-Weinberg equilibrium and linkage disequilibrium were performed for each population in Genepop v4.0^48^.

The autosomal Chd1 gene was amplified using two sets of primers (EX26F and EX27R; EMB26F and EMB27R)^49^ because some individuals failed to amplify with one set, and so we tried a second set. For the first set of primers, the PCR conditions were an initial denaturation at 95 °C for 5 min, followed by 36 cycles of denaturation at 94 °C for 30 s, annealing at 63 °C for 30 s, and extension at 70 °C for 2 min 30 s, and a final extension at 72 °C for 10 min. For the second set, the PCR conditions were 35 cycles of denaturation at 94 °C for 45 s, annealing at 59 °C for 45 s, and extension at 72 °C for 1 min. Heterozygous sites in Chd1 sequences were resolved to two haplotypes per individual using PHASE 2.1^50^ implemented in DnaSP v5^51^.

#### Genetic analyses

Haplotype diversity (h) and nucleotide diversity (π) were calculated for mitochondrial Cyt*b* and control region for each population and *mt* lineage. The uncorrected Cyt*b* genetic distances within and among *mt* lineages were calculated in MEGA 5.05^52^. For microsatellites, expected heterozygosity (H_e_) and observed herterozygosity (H_o_) were calculated using Genepop, and mean allele number and allelic richness for per locus and per taxon was assessed in Fstat 2.9.3^53^. For the Chd1 gene, π, H_e_ and H_o_ for each taxon was calculated using Arlequin v3.5^54^.

To examine the demographic history of each *mt* lineage, we only analyzed the control region due to its greater variability at this level. We used Tajima’s *D*^55^ and Fu’s *F*_*s*_ tests as implemented in Arlequin to test for neutrality. The goodness-of-fit of distributions was tested and its significant difference from a model of sudden expansion was assessed using the sum of squared deviations (SSD) and the raggedness index (r) with 1,000 parametric bootstrap replicates. Generally, low and non-significant SSD and r values indicate population expansion. For the expanding *mt* lineages, we estimated the time of expansion (t) from τ = 2ut, where τ is calculated as the time to expansion in mutational units and u is the mutation rate per generation for the entire sequence. The *u* is equal to *μgk*, where *μ* is the mutation rate per nucleotide, *g* is the generation time and *k* is the sequence length. We used a mutation rate of 20% ⁄ Myr for the current control region based on previous studies on bats^17^,^56^,^57^ and a generation time of 2 years based on data from a congeneric species^21^.

We calculated nuclear genetic distances between localities using Slatkin’s linearized F_ST_ (given by F_ST_/(1 − F_ST_)). An isolation by distance (IBD) model was estimated from the large forms based on microsatellites.

#### Phylogenetic analyses

For the mitochondrial analyses, two closely related species, *Rhinolophus mashalli* and *R. pusillus* were selected as outgroups (GenBank accession EU434938, EF217391, EU053160 and DQ642898). The nuclear Chd1 gene was not included in this analysis as part of the goal was to explore the different signals between the mitochondrion and the nuclear genome. Phylogenetic reconstruction using maximum likelihood (ML) in PhyML-aBayes^58^,^59^ and Bayesian Inference^49^ in MrBayes 3.1.2^60^ was undertaken for the Cyt*b* and control region separately as well as combined.

We used jModelTest^61^ and the Akaike information criterion to select the best model of evolution for ML and BI analyses. The best-fit models were as follows: (1) TrN + G [G = 0.072] for Cyt*b*, (2) TIM3 + I + G [I = 0.478; G = 0.507] for control region, TrN + I + G [I = 0.651; G = 0.714] for the combined mtDNA data. For ML analysis, the starting tree was obtained with BIONJ^62^ and we evaluated support of the resulting topologies using 1,000 nonparametric bootstraps. For BI analysis, we used the molecular evolution model parameters estimated for each data set and two simultaneous runs of Markov chain Monte Carlo (MCMC) analysis, each comprising four chains and 10^6^ generations. Trees and parameters were sampled every 10 generations. When the run terminated, the deviation of split frequencies reached a value <0.01. The ln-likelihoods of trees reached an asymptote. The first 25% of the sampled trees were discarded as a burn-in.

For the microsatellite analyses, we used a phylogenetic tree based on Nei *et al.*^63^ D_A_ distance between individuals and reconstructed using a NJ algorithm using Populations v1.2.30 64. Second, we used a Bayesian approach using structure v2.3.1^65^ on the microsatellite data. The admixture model without *a priori* designation for populations was used and the number of tested clusters (*K*) was based on the mtDNA results. For *K* = 1 to 5, 10 runs were performed with 500,000 iterations after 100,000 iterations were discarded as “burn-in”. The most likely number of populations was determined using the ΔK method described by Evanno *et al.*^66^. The graphical display of the genetic structure was produced by DISTRUCT^67^. Third, we used GENETIX v4.05.3 68 for factorial correspondence analysis (FCA) on the microsatellite data to further examine the degree of population substructuring among *R. macrotis* species complex. Fourth, we used GENELAND^69^ to determine the genetic boundaries between bat clusters based on *K* = 2 which is most likely according to STRUCTURE and FCA outputs. Five independent runs were performed for *K* = 2, using 10^7^ iterations with a burn-in of 30,000 iterations and 250 thinning. As the correlated model seems to be more prone to algorithm instabilities and more sensitive to departure from model assumptions, such as the presence of isolation by distance, we used the uncorrelated model of allele frequencies for microsatellite loci^70^. Consistency of the resulting inference was checked by comparing parameter estimates from 5 independent runs of GENELAND. For the Chd1 analysis, we used Network^71^ to construct a haplotype network for comparison of genealogical relationships among the haplotypes.

### Time of divergence

To estimate the time of divergence among clades, we only used Cyt*b* sequences because the molecular evolutionary rate of this gene is well-known for inter-clade and interspecific comparisons. The time to the most recent common ancestor (TMRCA) among lineages was estimated in BEAST v1.7.4^72^. A strict molecular clock was applied with a fixed mean substitution rate of 1.30 × 10^−8^ subs/site/year [calculated based on Nabholz *et al.*^73^; Nabholz, pers. com.; n = 223 Chiropteran species]. The TrN + I substitution model was used, as determined by AIC implemented in jModelTest. No outgroup was specified and the Yule process was used as a tree prior. UPGMA was used to construct the starting tree. The program was run for 5,000,000 generations (10% discarded as burn-in) and sampled every 1,000, which were then combined in Tracer version 1.4^74^. ESS values exceeded 2,000 for all parameters. All other initial parameters settings were the default provided by BEAST.

### Echolocation call recording and analysis

Echolocation calls were recorded with a D980 Pettersson bat detector (Pettersson Electronik AB, Uppsala, Sweden; frequency response 8 and 160 kHz ± 3.5 dB). This detector was positioned approximately 30 cm in front of hand-held bats. The recordings were transferred to a computer with a time expansion of ×10 using BatSound Pro 3.10 (sampling frequency of 441 kHz, 16 bit precision). The resting frequency that the bats emitted under these conditions is the stable constant frequency portion of the echolocation pulses emitted by the motionless bat. We selected a portion of a recording in which resting frequency calls were continuously uttered. Using criteria proposed by Sun *et al.*^30^, 5 high-quality calls were measured for the constant frequency component in the dominant second harmonic from power spectra of a call from each individual, and the mean value was used in the analysis.

### Phenotypic analyses

Geographic variation for phenotypic characters was explored via box plots, with longitude and latitude on the abscissa. The relationship of resting frequency to either longitude or latitude was estimated by linear regression analysis, with averaging of frequency by site.

To estimate the phenotypic differentiation between *mt* Clade 2 and *mt* Clade 3 in the large form, we used a general linear model with type III sums of squares to test for an effect of sex, site and *mt* lineage on forearm length and echolocation frequency. All variables were treated as fixed effects in the model.

## Additional Information

**Accession Codes**: All sequence data has been uploaded to GenBank (KX261888-KX261966, KX826057-KX826076). Microsatellite genotypes, sequence alignments by markers, phylogenetic trees and morphological and acoustic data files are uploaded on the Dryad Digital Repository (doi:10.5061/dryad.b1c88).

**How to cite this article**: Sun, K. *et al.* The complex evolutionary history of big-eared horseshoe bats (*Rhinolophus macrotis* complex): insights from genetic, morphological and acoustic data. *Sci. Rep.* **6**, 35417; doi: 10.1038/srep35417 (2016).

## Supplementary Material

Supplementary Information

## Figures and Tables

**Table 1:** Estimates of uncorrected genetic divergences (minimum-maximum values) for Cyt*b* gene within and between *mt* lineages (expressed as percent divergent).

	*mt* Clade 1	*mt* Clade 2	*mt* Clade 3
*mt* Clade 1	0.1–2.0		
*mt* Clade 2	2.4–3.2	0.1–1.6	
*mt* Clade 3	3.1–3.9	2.4–3.2	0.1–0.8

**Table 2:** Diversity estimates for different *mt* lineages of each form, based on the combined Cyt*b* and control region, control region and microsatellites.

		*mt* lineage	Small form	Large form	
			Clade 1	Clade 2	Clade 3
mtDNA	Cyt-b	N_ind_/N_h_	36/11	22/8	21/10
		h	0.83	0.85	0.91
		π(%)	1	0.83	0.37
	control region	N_ind_/N_h_	36/25	22/11	21/14
		h	0.97	0.87	0.94
		π(%)	4.24	4.09	2.83
ncDNA	microsatellites	N_ind_/A	32/12.25	46/14.875	
		A_R_	12.25	13.66	
		H_o_	0.71	0.76	
		H_e_	0.85	0.87	
	Chd1	N_ind_/N_h_	35/9	36/12	
		π(%)	0.15	0.16	
		H_o_	0.05	0.08	
		H_e_	0.09	0.10	

Number of individuals (N_ind_), number of haplotypes (N_h_), haplotype diversity (h), nucleotide diversity (π), mean allele number (A), allelic richness (A_R_), expected heterozygosity (H_o_) and observed heterozygosity (H_e_).

**Table 3:** Mean estimates of time to most recent common ancestor (TMRCA) and 95% credible intervals for each *mt* lineage based on Cyt*b* gene.

Node	TMRCA (Ma)	CI (95%)
*mt* Clade 1 (small forms)	0.64	0.44–0.85
*mt* Clade 2 (large forms)	0.66	0.43–0.89
*mt* Clade 3 (large forms)	0.33	0.19–0.49
*mt* Clades 1 + 2	1.16	0.87–1.47
*mt* Clades 1 + 2 + 3 (all)	1.51	1.14–1.88

Analyses were performed using a molecular clock rate of 1.3% per million years.

**Table 4:** Results of Fu’s Fs test, Tajima’s D test, mismatch distribution analysis and estimation of the time of population expansion for *Rhinolophus macrotis* complex based on control region.

	Fu’s Fs	Tajima’s D	SSD	r	Tao (95% CI)	T_MD_(95% CI) k_a_
Clade 1	−1.643	0.371	0.008	0.008	29.207 (15.191–36.018)	78.7 (40.9–97.0)
Clade 2	3.949	0.760	0.043**	0.077**	—	—
Clade 3	−0.482	0.599	0.026**	0.077**	—	—

Statistically significant results are indicated by asterisks: ^*^P < 0.05, ^**^P < 0.01.

**Table 5:** Morphological and acoustic differentiation of large forms in *Rhinolophus macrotis* complex (i.e. *mt* Clade 2 + *mt* Clade 3) according to *mt* lineage, sex and site, analyzed by general linear model (GLM).

Factor	Forearm length					Resting frequency				
	d.f.	Sum Sq	Mean Sq	F value	*P*	d.f.	Sum Sq	Mean Sq	F value	*P*
Sex	1	0.038	0.038	0.008	0.931	1	36.345	36.345	11.953	0.001*
*mt* lineage	1	11.326	11.326	2.253	0.139	1	153.082	153.082	50.347	0.000*
Sex × *mt* lineage	1	4.990	4.990	0.992	0.324	1	3.907	3.907	1.285	0.262
Error	53	266.486	5.028			53	161.147	3.041		
Factor	d.f.	Sum Sq	Mean Sq	F value	*P*	d.f.	Sum Sq	Mean Sq	F value	*P*
Site	8	75.831	9.479	2.255	0.040*	8	172.740	21.593	33.240	0.000*
*mt* lineage	1	2.582	2.582	0.614	0.437	1	0.805	0.805	1.239	0.271
Site × *mt* lineage	1	1.698	1.698	0.404	0.528	1	0.013	0.013	0.020	0.888
Error	46	193.347	4.203			46	29.882	0.650		
