# Supplementary material for: The complex evolutionary history of big-eared horseshoe bats (Rhinolophus macrotis complex): insights from genetic, morphological and acoustic data
Source: Sci Rep. 2016 Oct 17;6:35417. doi: 10.1038/srep35417 (PMC5066199; doi:10.1038/srep35417)
Supplement: Supplementary Information [file srep35417-s1.pdf]

Supplementary Information

The complex evolutionary history of big-eared horseshoe bats (*Rhinolophus macrotis* complex): insights from genetic, morphological and acoustic data

Keping Sun[1], Rebecca T. Kimball[2], Tong Liu[1], Xuewen Wei[1], Longru Jin[1], Tinglei Jiang[1], Aiqing Lin[1], and Jiang Feng[1,*]

[1]*Jilin Provincial Key Laboratory of Animal Resource Conservation and Utilization, Northeast Normal University, Changchun, China*

[2]*Department of Biology, University of Florida, Gainesville, Florida, United States of America*

[*]*Corresponding author. E-mail: fengj@nenu.edu.cn*

Telephone and fax number: +86-431-85099866

Table S1 Sample information.

Colony	Sampling locality	Longititude	Latitude	Nmt	cytb haplotype	control region haplotype	Nnc	Chd1 haplotype
**small form (mt Clade 1)**				36			35	
**GD1**	Yangchun, Guangdong Province	111.94	22.44	1	H1	H1	1	H1, H20
**GD2**	Shaoguan, Guangdong Province	113.56	24.77	10	H2	H2-H9	9	H2, H20
**GX**	Nanning, Guangxi Province	108.70	22.82	5	H3	H10-H13	5	H2, H20
**HuN**	Lengshuijiang, Hunan Province	111.57	27.75	3	H2	H14	3	H2, H20
**JX1**	Jianggangshan, Jiangxi Province	114.20	26.60	1	H2	H15	4	H2, H3, H20
**YN1**	Jinning, Yunnan Province	102.33	24.50	4	H4-H6	H16-H18	4	H7, H8, H20
**YN2**	Yuanjiang, Yunnan Province	102.00	23.59	7	H7, H8	H19- H21	5	H2, H20
**YN3**	Baoshan, Yunnan Province	99.10	25.08	5	H9, H10	H22-H24	4	H4-H8, H20
**large form (mt Clade 2)**				22			19	
**CQ**	Wulong, Chongqing City	107.95	29.27	2	H11	H25	2	H12, H18, H20
**GD1**	Yangchun, Guangdong Province	111.94	22.44	6	H12, H13	H26, H27	6	H14-H18
**GZ**	Xishui, Guizhou Province	106.21	28.45	4	H14-H16	H28-H31	3	H10, H11, H18, H20
**SC**	Dazhou, Sichuan Province	107.74	31.20	6	H17, H18	H32, H33	6	H10, H11, H18-H20
**SXi**	Hanzhong, Shannxi Province	107.03	32.84	3	H18, H19	H32-H35	2	H9, H18, H20
**HeN**	Xichuan, Henan Province	111.55	32.87	1	H19	H36	0	—
**large form (mt Clade 3)**				21			17	
**CQ**	Wulong, Chongqing City	107.95	29.27	1	H20	H37	1	H18, H20
**HuN**	Lengshuijiang, Hunan Province	111.57	27.75	5	H21-H24	H38-H42	6	H15, H17, H18, H20
**JX1**	Jianggangshan, Jiangxi Province	114.20	26.60	6	H25, H26	H43-H46	4	H15, H17, H18
**JX2**	Ganzhou, Jiangxi Province	114.09	25.46	5	H27, H28	H47- H49	2	H18, H20
**SXi**	Hanzhong, Shannxi Province	107.03	32.84	4	H29	H50	4	H9, H18

Nmt, the number of mitochondrial DNA sequences. Nnc, the number of nuclear Chd1 gene sequences.

Table S2 Diversity estimates for each form. Number of individuals (N), mean allele number (A), allelic richness (A_R), expected heterozygosity (H_o) and observed heterozygosity (H_e).

Taxon	Populations	N	A	A_R	H_E	H_O
large form	GZ	4.0	5.38	1.86	0.86	0.84
	CQ	4.0	5.25	1.87	0.87	0.72
	SC	6.0	5.75	1.81	0.81	0.77
	SiX	7.0	5.75	1.78	0.78	0.71
	HeN	2.0	——	——	0.81	0.69
	JX1	5.0	6.00	1.85	0.85	0.83
	JX2	6.0	6.50	1.86	0.86	0.83
	GD2	6.0	4.38	1.71	0.71	0.67
	HuN	6.0	6.25	1.85	0.85	0.77
small form	HuN	3.0	3.75	1.80	0.80	0.75
	YN1	4.0	5.13	1.81	0.81	0.81
	YN2	5.0	5.25	1.80	0.80	0.73
	YN3	5.0	4.25	1.69	0.69	0.58
	GX	5.0	4.38	1.74	0.74	0.75
	GD1	9.0	4.38	1.69	0.69	0.71
	GD2	1.0	——	——	——	——

Table S3 Resting frequency (RF) in echolocation calls and forearm length (FA) of the large form and small form in the *R. macrotis* complex populations from China.

**Taxon and colony**	**mRF(kHz)**			**FA(mm)**		
	Nf/Nm	female	male	Nf/Nm	female	male
**large form (*mt* Clade 2)**						
**CQ**	0/2	——	50.05	0/2	——	44.84
**GD1**	2/4	52.78	51.66	2/4	48.21	46.47
**GZ**	0/4	——	49.33	0/4	——	45.63
**SC**	2/4	49.80	49.86	2/4	45.22	47.51
**SXi**	3/0	52.82	——	3/0	46.71	——
**HeN**	0/2	——	54.44	0/2	——	44.95
**large form (*mt* Clade 3)**						
**CQ**	0/1	——	49.32	0/1	——	46.94
**HuN**	10/12	46.52	46.16	10/12	48.45	47.25
**JX1**	9/2	49.39	46.80	9/2	45.17	48.00
**JX2**	4/2	47.64	46.50	4/2	49.60	49.62
**SXi**	4/0	52.26	——	4/0	——	46.93
**Small form (*mt* Clade 1)**						
**GD1**	0/0	——	——		——	39.57
**GD2**	8/3	69.01	67.79	12/3	40.12	38.50
**GX**	1/2	67.76	66.29	2/3	39.75	39.67
**HuN**	2/1	67.12	66.06	2/1	39.95	39.69
**JX1**	2/0	64.70	——	2/0	39.75	——
**YN1**	3/9	57.83	57.41	6/14	43.42	41.93
**YN2**	0/0	——	——	3/7	40	39.14
**YN3**	0/0	——	——	11/1	43.05	45

Fig. S1 Phylogenetic tree based on the analysis of the mitochondrial Cyt*b* of the *R. macrotis* complex. Numbers above the branches are the bootstrap support obtained in maximum likelihood and posterior probabilities from Bayesian analyses.

Fig. S2 Phylogenetic tree based on the analysis of the mitochondrial control region of the *R. macrotis* complex. Numbers above the branches are the bootstrap support obtained in maximum likelihood and posterior probabilities from Bayesian analyses.

Fig. S3 Output from Geneland showing assignments to clusters for K = 2. Black dots represent localities analysed in this study. In five independent runs, three runs resulted in the clusters of (a) and (b), while two runs resulted in the clusters of (c) and (d).

Fig. S4 Phylogenetic tree based on the analysis of the mitochondrial Cyt*b* of all the *R. macrotis* complex and *Rhinolophus sp1.* (EU434937). Numbers above the branches are the bootstrap support obtained in maximum likelihood analyse.

Fig. S1

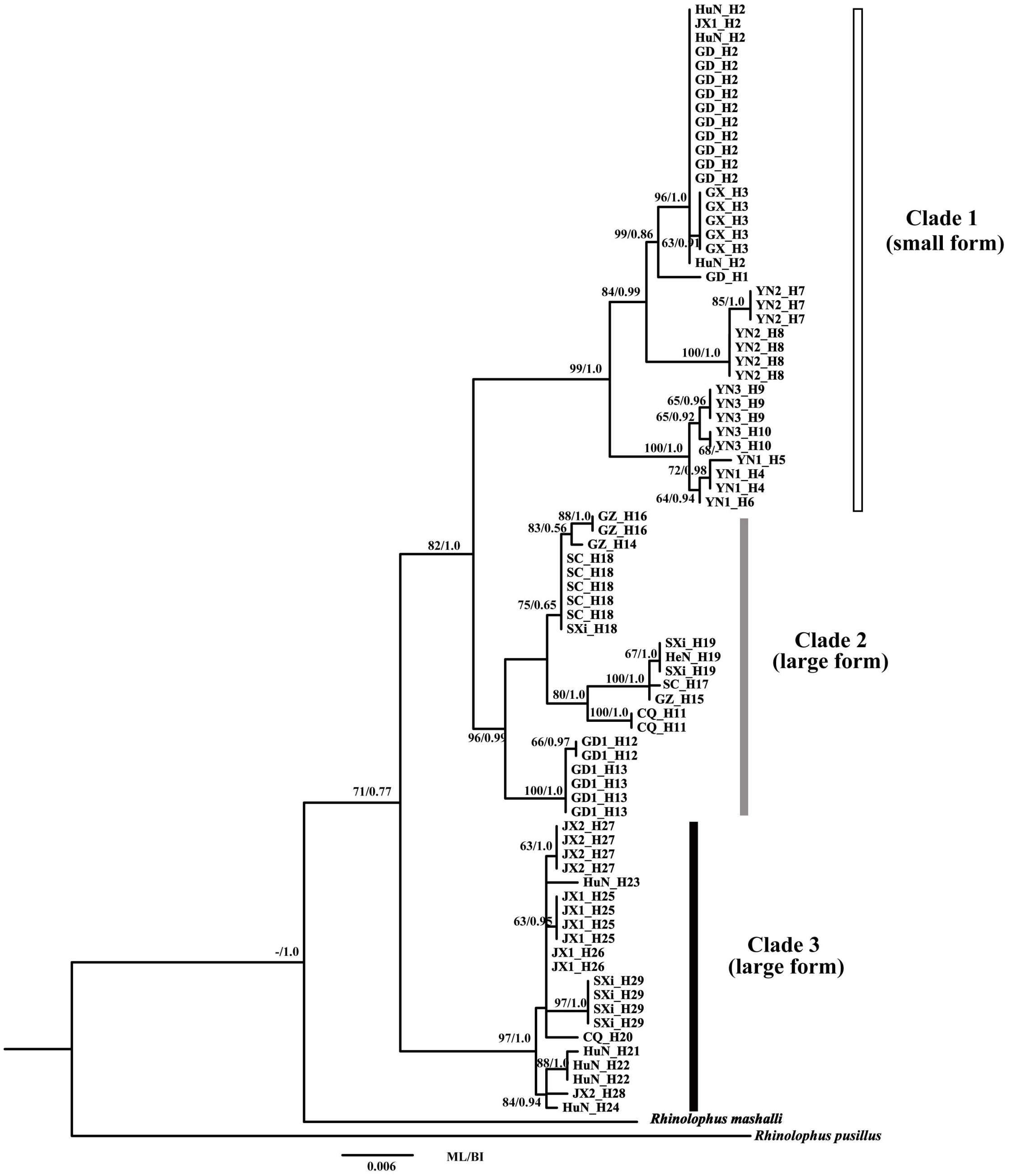

HuN_H2
JX1_H2
HuN_H2
GD_H2
GD_H2
GD_H2
GD_H2
GD_H2
GD_H2
GD_H2
GD_H2
GD_H2
GD_H2
96/1.0
GX_H3
GX_H3
GX_H3
99/0.86
63/0.91
GX_H3
GX_H3
HuN_H2
GD_H1
84/0.99
YN2_H7
85/1.0
YN2_H7
YN2_H7
YN2_H8
100/1.0
YN2_H8
YN2_H8
YN2_H8
99/1.0
YN3_H9
65/0.96
YN3_H9
65/0.92
YN3_H9
YN3_H10
100/1.0
YN3_H10
68/-
YN1_H5
72/0.98
YN1_H4
YN1_H4
64/0.94
YN1_H6
88/1.0
GZ_H16
83/0.56
GZ_H16
GZ_H14
82/1.0
SC_H18
SC_H18
SC_H18
SC_H18
75/0.65
SC_H18
SXi_H18
SXi_H19
67/1.0
HeN_H19
SXi_H19
100/1.0
SC_H17
80/1.0
GZ_H15
100/1.0
CQ_H11
CQ_H11
96/0.99
66/0.97
GD1_H12
GD1_H12
GD1_H13
GD1_H13
100/1.0
GD1_H13
71/0.77
GD1_H13
JX2_H27
JX2_H27
63/1.0
JX2_H27
JX2_H27
HuN_H23
JX1_H25
JX1_H25
63/0.95
JX1_H25
JX1_H25
-/1.0
JX1_H26
JX1_H26
SXi_H29
SXi_H29
97/1.0
SXi_H29
SXi_H29
97/1.0
CQ_H20
HuN_H21
88/1.0
HuN_H22
HuN_H22
JX2_H28
84/0.94
HuN_H24
Rhinolophus mashalli
Rhinolophus pusillus
0.006
ML/BI
Clade 1
(small form)
Clade 2
(large form)
Clade 3
(large form)

Fig. S2

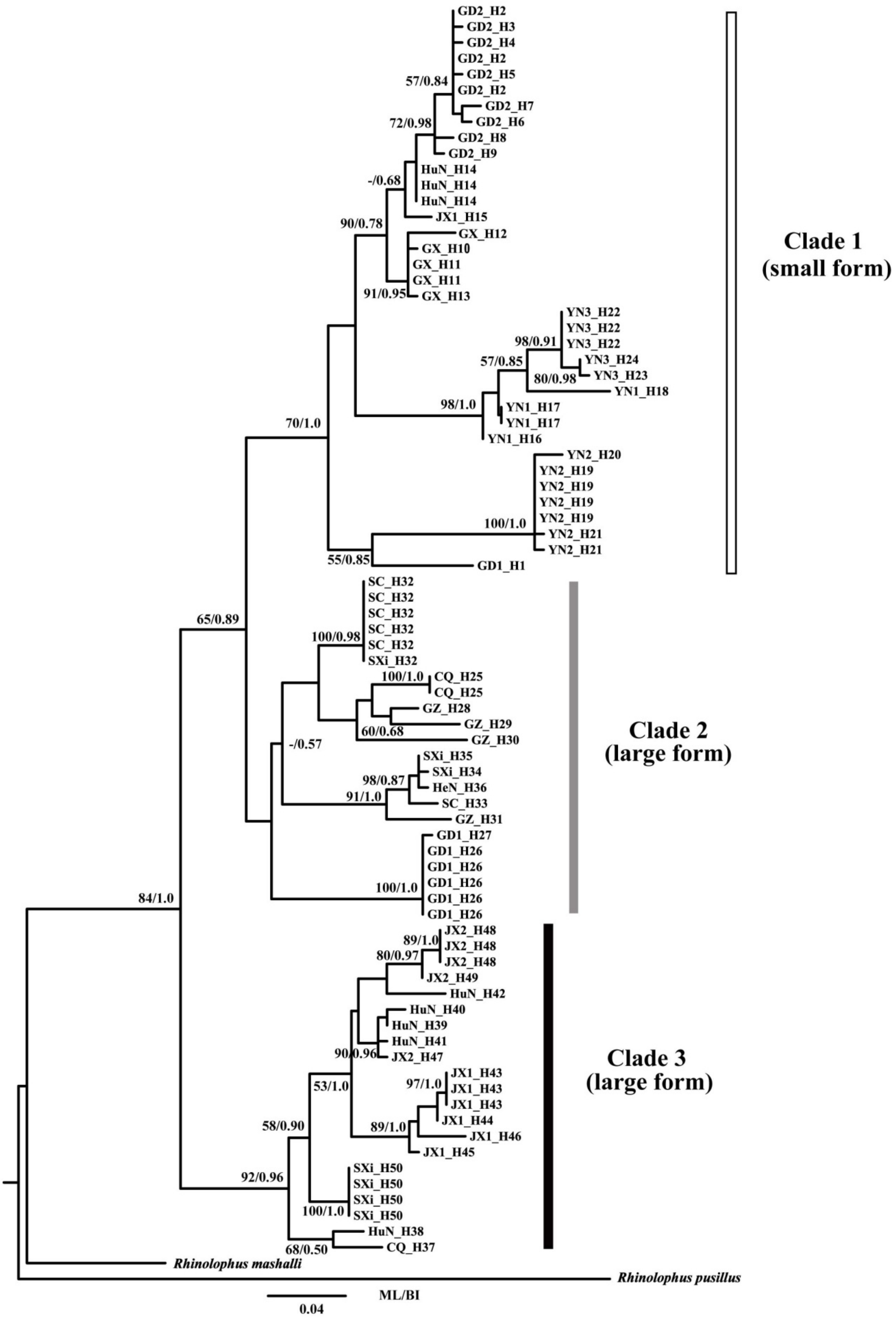
GD2_H2
GD2_H3
GD2_H4
GD2_H2
GD2_H5
GD2_H2
GD2_H7
GD2_H6
GD2_H8
GD2_H9
57/0.84
72/0.98
-/0.68
HuN_H14
HuN_H14
HuN_H14
JX1_H15
90/0.78
GX_H12
GX_H10
GX_H11
GX_H11
GX_H13
91/0.95
YN3_H22
YN3_H22
YN3_H22
YN3_H24
YN3_H23
YN1_H18
98/0.91
57/0.85
80/0.98
98/1.0
YN1_H17
YN1_H17
YN1_H16
70/1.0
YN2_H20
YN2_H19
YN2_H19
YN2_H19
YN2_H19
YN2_H21
YN2_H21
100/1.0
55/0.85
GD1_H1
Clade 1
(small form)
SC_H32
SC_H32
SC_H32
SC_H32
SC_H32
SXi_H32
100/0.98
65/0.89
100/1.0
CQ_H25
CQ_H25
GZ_H28
GZ_H29
GZ_H30
60/0.68
-/0.57
SXi_H35
SXi_H34
HeN_H36
SC_H33
GZ_H31
98/0.87
91/1.0
GD1_H27
GD1_H26
GD1_H26
GD1_H26
GD1_H26
GD1_H26
100/1.0
84/1.0
Clade 2
(large form)
JX2_H48
JX2_H48
JX2_H48
JX2_H49
HuN_H42
89/1.0
80/0.97
HuN_H40
HuN_H39
HuN_H41
JX2_H47
90/0.96
JX1_H43
JX1_H43
JX1_H43
JX1_H44
JX1_H46
JX1_H45
97/1.0
53/1.0
89/1.0
58/0.90
SXi_H50
SXi_H50
SXi_H50
SXi_H50
92/0.96
100/1.0
HuN_H38
CQ_H37
68/0.50
Clade 3
(large form)
Rhinolophus mashalli
Rhinolophus pusillus
ML/BI
0.04

Fig. S3

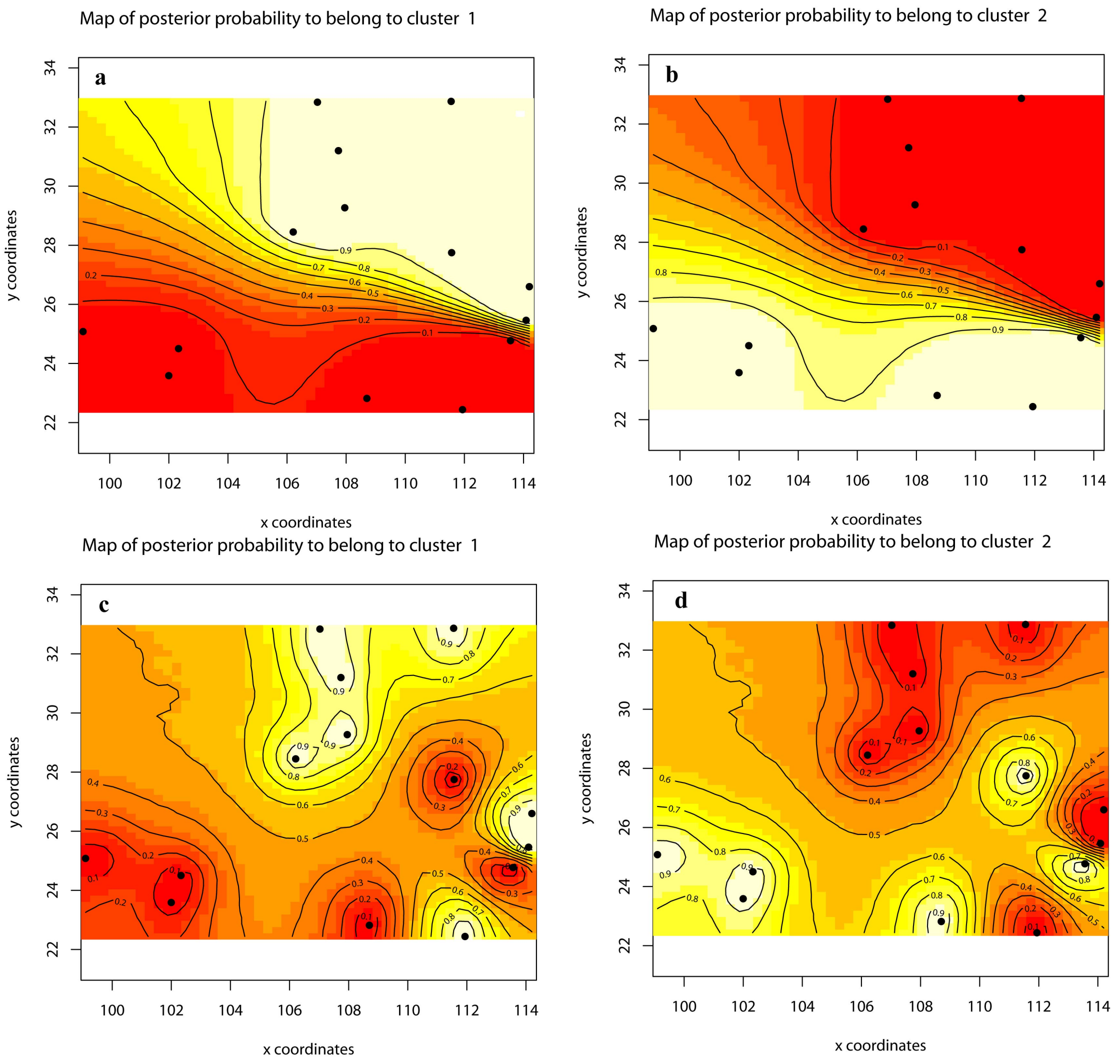
Map of posterior probability to belong to cluster 1
a
y coordinates
x coordinates
Map of posterior probability to belong to cluster 2
b
y coordinates
x coordinates
Map of posterior probability to belong to cluster 1
c
y coordinates
x coordinates
Map of posterior probability to belong to cluster 2
d
y coordinates
x coordinates

Fig. S4

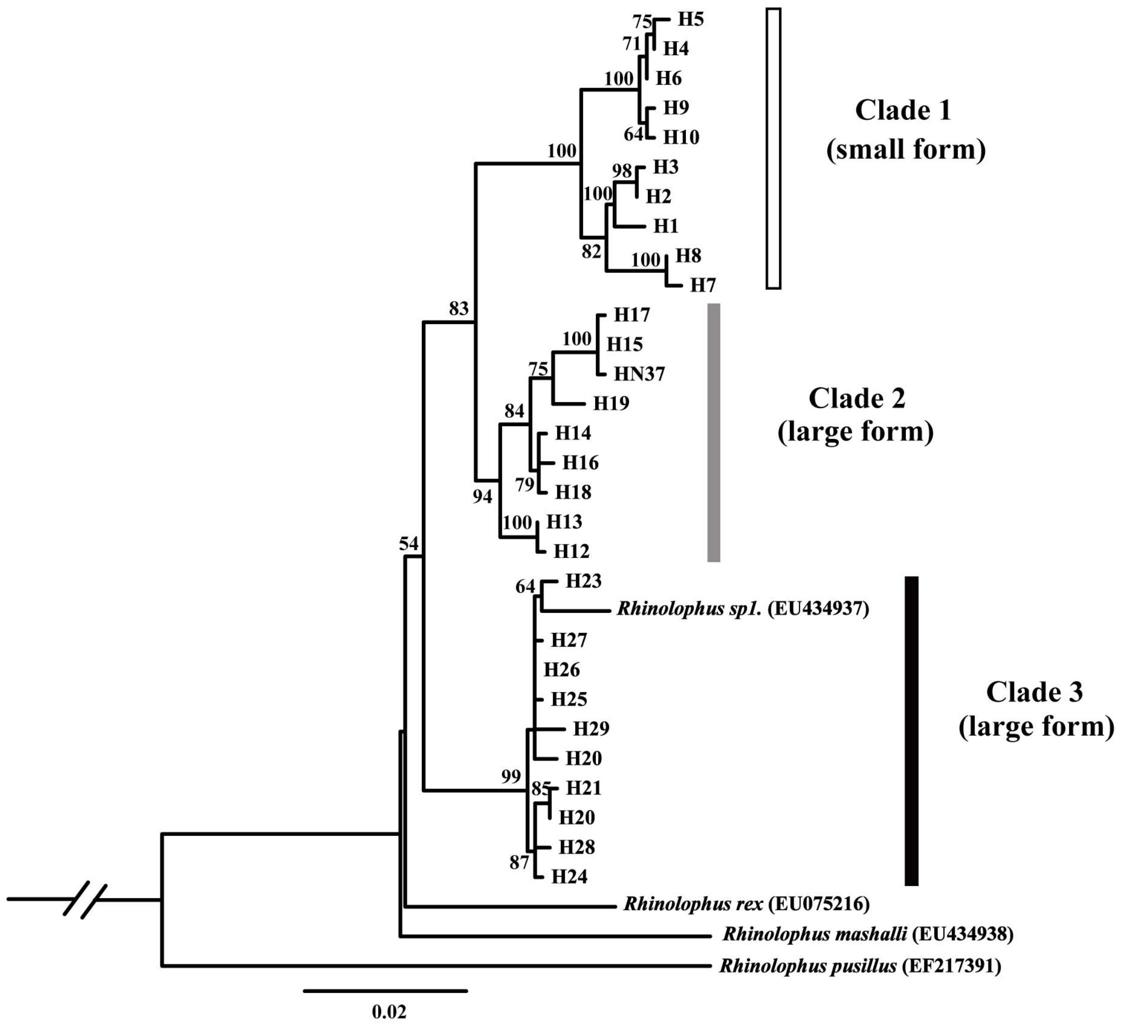
H5
H4
H6
H9
H10
H3
H2
H1
H8
H7
H17
H15
HN37
H19
H14
H16
H18
H13
H12
H23
Rhinolophus sp1. (EU434937)
H27
H26
H25
H29
H20
H21
H20
H28
H24
Rhinolophus rex (EU075216)
Rhinolophus mashalli (EU434938)
Rhinolophus pusillus (EF217391)
75
71
100
64
100
98
100
82
100
83
100
75
84
79
94
100
54
64
99
85
87
Clade 1
(small form)
Clade 2
(large form)
Clade 3
(large form)
0.02
